# An Aligned-Gap and Centered-Gap Rectangular Multiple Split Ring Resonator for Dielectric Sensing Applications

**DOI:** 10.3390/s140713134

**Published:** 2014-07-21

**Authors:** Izyani Mat Rusni, Alyani Ismail, Adam Reda Hasan Alhawari, Mohd Nizar Hamidon, Nor Azah Yusof

**Affiliations:** 1 Wireless and Photonic Network Research Centre, Faculty of Engineering, Universiti Putra Malaysia, Selangor 43400, Malaysia; E-Mails: alyani@upm.edu.my (A.I.); adamreda@upm.edu.my (A.R.H.A.); 2 Institute of Advanced Technology, Universiti Putra Malaysia, Selangor 43400, Malaysia; E-Mails: mnh@upm.edu.my (M.N.H.); azahy@upm.edu.my (N.A.Y.)

**Keywords:** sensor, metamaterial, split ring resonator, dielectric sensing

## Abstract

This paper presents the design and development of a planar Aligned-Gap and Centered-Gap Rectangular Multiple Split Ring Resonator (SRR) for microwave sensors that operates at a resonance frequency around 5 GHz. The sensor consists of a microstrip transmission line loaded with two elements of rectangular SRR on both sides. The proposed metamaterial sensors were designed and fabricated on Rogers RT5880 substrate having dielectric constant of 2.2 and thickness of 0.787 mm. The final dimension of the proposed sensor was measured at 35 × 14 mm^2^. Measured results show good agreement with simulated ones as well as exhibiting high *Q*-factor for use in sensing application. A remarkably shift of resonance frequency is observed upon introduction of several sample with different dielectric value.

## 1. Introduction

Metamaterials are artificial materials synthesized by embedding specific metallic inclusions. Some of these materials exhibit either negative permittivity or negative permeability at resonance [1]. In recent years, numerous researchers have been attracted to design microwave sensors based on metamaterials due to their cost effectiveness, miniaturization and label-free detection capabilities. Schubler *et al.* [2] reviewed metamaterial-inspired composite right/left handed transmission line microwave sensors. Chen *et al.* [3] reviewed metamaterial applications in sensing with emphasis on split-ring resonator (SRR)-based sensors. Huang *et al.* [4] studied the performance of microwave sensors using metamaterials, followed by Yang *et al.* [5], and the results showed that the sensitivity of the sensors can be greatly enhanced by using metamaterials. Recently, several sensors based on modified SRRs have been successfully introduced for displacement and velocity detection applications [6,7]. Furthermore, a diamond-shape tapered SRR [8], and a horn-shaped SRR [9] were proposed to have a fixed resonant frequency so the sensor can be operated as an inexpensive single frequency system.

Generally, planar metamaterials which consists of a subwavelength resonator can exhibit a strong localization and enhancement of fields to improve the sensor selectivity for detecting nonlinear substances and enable detection of extremely small amounts of substrate [10]. Among the many metamaterial unit cell designs, metal split ring resonators with different configurations have been extensively demonstrated experimentally and successfully applied in biomolecular sensing. In earlier designs, several double SRRs (DSRR) or single SRRs have been used. For example, Lee *et al.* [11,12] proposed a single element planar DSRR-based biosensor to detect DNA hybridization. In this design, the biomolecule is made to bind to the surface of the ring by immersing the sensor into several solutions which might degrade the performance of the sensor itself.

Wiwatcharagoses *et al.* [13] had introduced a modified split ring resonator to be implemented in near field sensing of dielectric materials. The sensor consists of a microstrip line loaded with circular SRRs on both sides that are directly connected to the edge of the line. The issue is when depositing the sample at several places, this introduces a high degree of inaccuracy and yet the idea of covering the whole area is not practical when the sample availability or the amount is very limited. Another group of researchers had employed microfluidics channels on metamaterial-based sensors in order to deliver liquid-type samples onto the sensing area [14,15]. However, some of the designs showed degradation in the robustness and sensitivity of the sensor when microfluidics channel is introduced.

An SRR can be approximated by an inductor and capacitor in the form of a series *LC* resonant circuit. Specifically, the ring forms the inductor and the split forms the capacitor. The resonance takes place in the SRR when the electric energy stored in the capacitor is balanced with the magnetic energy stored in the inductor. The changes in capacitance or inductance due to dielectric loading from dielectric samples leads to a considerable shift in the resonance frequency, *f_r_* which is given by Equation (1). Measurement in delta shift in resonance frequency allows direct sensing of small volume of sample:

(1) $$f_{r}=\frac{1}{2\pi \sqrt{LC}}$$

To achieve higher sensitivity, the sensor needs to have a sharp resonance in frequency response and high concentration of electric field is require to enable the detection in dielectric environments when the interrogation sample is deposited on the surface of the resonator [16]. Generally, the field confinement of conventional SRRs is relatively weak, and bigger in size thus limiting its sensitivity in detecting any dielectric change [17]. As mentioned in [18], an increase in the split gap number would increase the high-field region, increasing the small volume of the dielectric probe and decreasing the operational frequency.

In this work, by adopting multi-ring with multiple gap rectangular SRRs onto a microstrip transmission line, a new structure has been constructed to the enhance sensing performance of the device. It is believed that by increasing a number of split gaps, it will provide a strong and localized field enhancement in the selected area and decrease the operating frequency. Furthermore, by introducing an extended split length, the capacitive region will be enlarged and electric field confinement will be increased. In order to improve the sensitivity of the SRR sensor, a device that has sharper and deeper dip in its transmission resonance, a much lower operating frequency and more regions of high electric fields will be selected.

## 2. Resonator Type for Material Characterization

### 2.1. Sensor Theory

Generally, the resonance method usually provides higher accuracy and better sensitivity compared to the non-resonant method. The resonance frequency changes when a sample interacts with the field distribution of a resonating structure. The change in resonance frequency and material properties of sample are related to each other and this can be expressed by the following Equation (2) [19]:

(2) $$\frac{\Delta f_{r}}{f_{r}}=\frac{\int_{v}\left(\Delta \in E_{0}\cdot E_{1}+\Delta \mu H_{0}\cdot H_{1}\right)dv}{\int_{v}\left(\Delta \in {\left|E_{0}\right|}^{2}+\Delta \mu {\left|H_{0}\right|}^{2}\right)dv}$$

where *Δf_r_* is the shift in the resonance frequency *f*r, Δε and *Δμ* are the change in the permittivity and permeability, respectively, and *v* is the perturbed volume. ***E****_0_* and ***H****_0_* are the field distributions without the perturbation and ***E****_1_* and ***H****_1_* are the field distributions with the perturbation. This equation shows that as the permittivity, ε, or permeability, μ, of a sample change, the frequency shift with respect to the based resonance frequency will also be changed. To date, the above mentioned perturbation approach were successfully employed by several researchers to extract the properties of the material [20,21].

### 2.2. Sensor Design

The proposed sensor is designed to study the feasibility of using metamaterials for dielectric material detection. Motivated from the research conducted by Bilotti [22] an aligned-gap multi-ring SRR structure is proposed as a transducer for dielectric sample detection. The basic idea in implementing the multi-ring SRR is to increase the distributed capacitance between the strips without increasing the area occupied by the resonator, leading to lower resonance frequency [23]. Moreover, the proposed aligned-gap multi-ring SRR (A-G MSRR) structure is designed in such a way so as to increase the high field region at the split and decrease the operating frequency due to the increment in capacitance value. A centered-gap multiple SRR (C-GMSRR) is proposed as a second design, where it combines multiple SRRs in a compact design, thus being able to miniaturize the whole structure [24].

**Figure 1.**
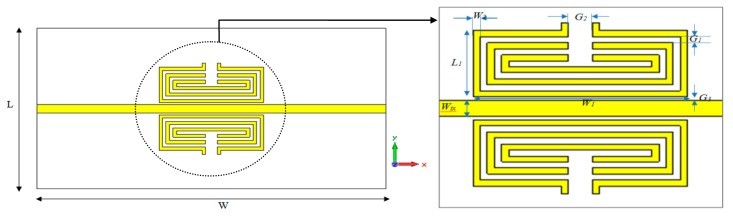
Schematic view of the aligned-gap MSRR structure and its dimensions.

**Figure 2.**
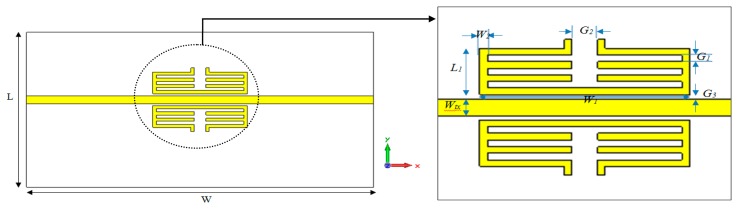
Schematic view of the centered-gap MSRR Structure and its dimensions.

Each one of the unit cells is composed of two identical A-GMSRR or C-GMSRR structures loaded with a microstrip transmission line as illustrated in Figures 1 and 2. When a resonator element is introduced on both sides, the proposed design has slightly lower resonant frequency and deeper dip in the transmission resonance. These resonators are excited by the time-varying *H*-Field of the microstrip line. For this design, the resonator is positioned in close proximity to a microstrip transmission line, which builds up a magnetic field around itself in a quasi-TEM wave propagation. This leads to an induced resonating current in the loop and generates an equivalent dipole moment which exhibits negative electromagnetic permeability. Furthermore, the distance between microstrip lines and resonators should be as small as possible to enhance the coupling. Therefore, a rectangular geometry is chosen instead of a circular ones to maximize coupling to the microstrip transmission line, which leads to stronger resonance of the device. The unit cell is designed with extended splits to interact with pertuber for which the dielectric changes are detected.

Based on the layout and proposed unit cell structures based on Figures 1 and 2, the sensors are designed to operate near 5 GHz using a full wave electromagnetic simulator [25]. The proposed sensors are designed on a planar substrate, a commercially available Rogers RT5880 substrate having a dielectric constant of 2.2 and thickness of 0.787 mm. The metallic inclusions are made of copper with the thickness of 0.035 mm and a conductivity of 5.8 × 10^7^ S/m and the substrate size is 35 × 14 mm. Table 1 summarizes the optimized parameters of each proposed structure.

**Figure 3.**
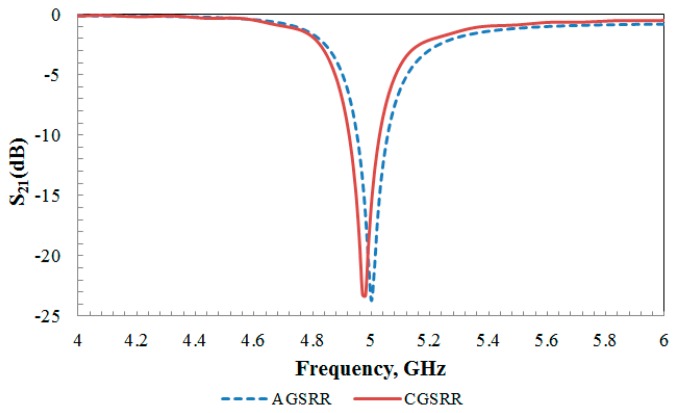
Simulated S-parameter for the aligned-gap MSRR and centered-gap MSRR.

Each of the proposed unit cells were simulated using a full-wave electromagnetic simulator [25] to obtain the transmission coefficients, S_21_ magnitudes. It is clearly observed that the simulated S_21_ for each structure in Figure 3 revealed a frequency at 5 GHz for the aligned-gap SRR and 4.98 GHz for the centered-gap SRR structure. The resonance dip for the aligned-gap and centered-gap structures are 23.70 dB and 23.30 dB, respectively.

### 2.3. Retrieving the Effective Parameters

In this paper, a modified Nicolson-Ross-Weir approach [26] was chosen to extract the permittivity and permeability from the S-parameter obtained using [25]. Equations (3) and (4) were used to determine the effective parameters, where *μ_r_* is the relative permeability and ε*_r_* is the relative permittivity:

(3) $$\mu_{r}=\frac{2}{jk_{0}d}*\frac{1-v_{2}}{1+v_{2}}$$

(4) $$\varepsilon_{r}=\frac{2}{jk_{0}d}*\frac{1-v_{1}}{1+v_{1}}$$

where *k_o_* is wave number and *d* is the thickness of substrate, while *v_1_* and *v_2_* are the composite terms of addition and subtraction of S-parameter and can be represented through Equations (5) and (6):

(5) $$v_{1}=S_{21}+S_{11}$$

(6) $$v_{2}=S_{21}-S_{11}$$

**Figure 4.**
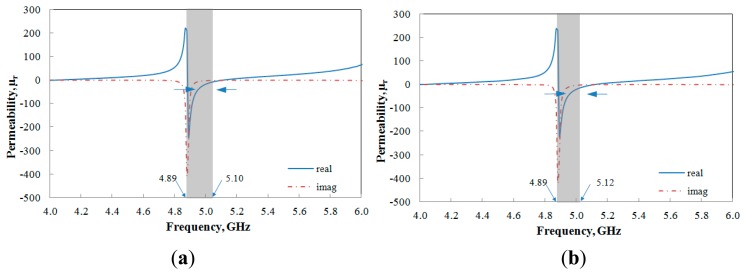
Effective parameters for (**a**) aligned-gap; (**b**) centered-gap sensor.

The retrieval results are shown in Figure 4. From the figure, these proposed structures exhibit real negative permeability at certain respective frequencies which indicates that these structures are μ negative metamaterials. It is obviously seen that the value of permeability (*μ_r_*) falls between 4.89 and 5.10 GHz for the aligned-gap multi-ring and 4.89 to 5.12 GHz for the centered-gap SRR structure within a frequency region as shown in Table 2.

## 3. Fabrication and Experimental Setup

### Sensor Fabrication

**Figure 5.**
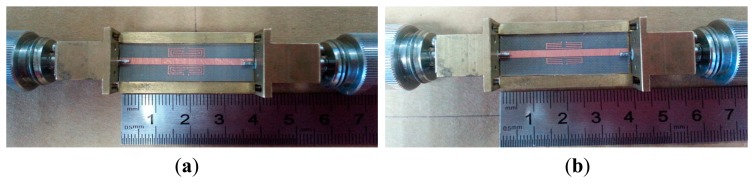
Photograph of the fabricated sensors: (**a**) aligned-gap; (**b**) centered-gap.

**Figure 6.**
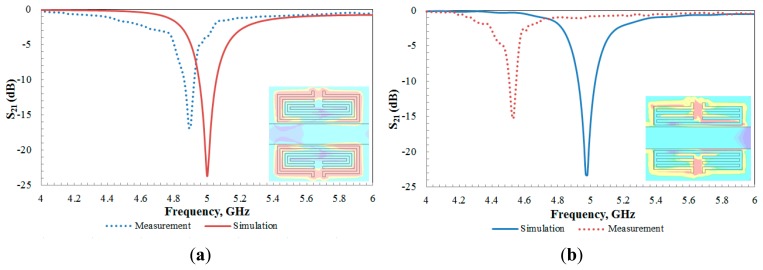
Simulated and measured S_21_ with (inset) E-field at 5 GHz for (**a**) aligned-gap; (**b**) centered-gap sensor.

The proposed sensors were fabricated on Roger RT5880 substrate according to the dimensions stated in Table 1 using standard photolithography techniques. The photograph of the fabricated sensor is shown in Figure 5. The sensor is formed on one side of the substrate, while the other side is grounded. A rectangular block of brass with SMA ports is used as the sensor holder to avoid misalignment by the coaxial cable when the sensor is directly connected to an Anritsu 37347D Vector Network Analyzer (VNA) for S-parameter measurements. For comparison purposes, the simulated and measured S_21_ for both designs are shown in Figure 6. Table 3 summarizes the simulated and measured responses.

As can be seen from the simulation graph in Figure 6, it obviously demonstrates that the aligned-gap structure has a narrower band and sharper dip which reveals its high *Q* nature compared with other structures. The *Q* factor of a resonance peak or dip can be calculated as in Equation (7) where Δ*f* is the bandwidth at +3 dB with respect to the minimal transitions [11,12]:

(7) $$Q=\frac{f_{0}}{\Delta f}$$

From the results, it can be concluded that all devices exhibit band stop transmission characteristics where the resonance frequency is relying on the size and type of the metamaterial unit cell. It is clear that the results show some small deviations between simulation and the measurement. The measured resonance frequencies for both sensors are slightly shifted from the simulation while the measured magnitude of transmission coefficient, S_21_, is slightly lowered than the simulated one. The difference between the simulated and the measured results is mainly due to mismatch between the SMA connectors and the feed lines as well as fabrication tolerance and limits in the simulation accuracy. Furthermore, the effects of the sample holder as well as the possibility of having a thin air layer between the sample holder and the sensor itself are excluded in the simulation for both sensors.

In order to adopt the proposed structure for bio-sensing applications, an electric field within the split gap needs to be observed. Generally, SRR structures are predicted to develop an intense and localized electric field within the split in the ring which is very sensitive to any dielectric. As illustrated in Figure 6 in the inset, on resonance, a strong electric field is established across the split gap. It is obviously seen that the aligned-gap SRR structure strongly confines the electric field around the capacitive gap, with peak values of 2.476 × 10^5^ V/m, which is higher than the centered-gap one with an electric field peak value of 2.167 × 10^4^ V/m, respectively, as expected. The reason behind this is due to the strong coupling across the gaps of the structure, which depends on the distance between the gaps.

**Figure 7.**
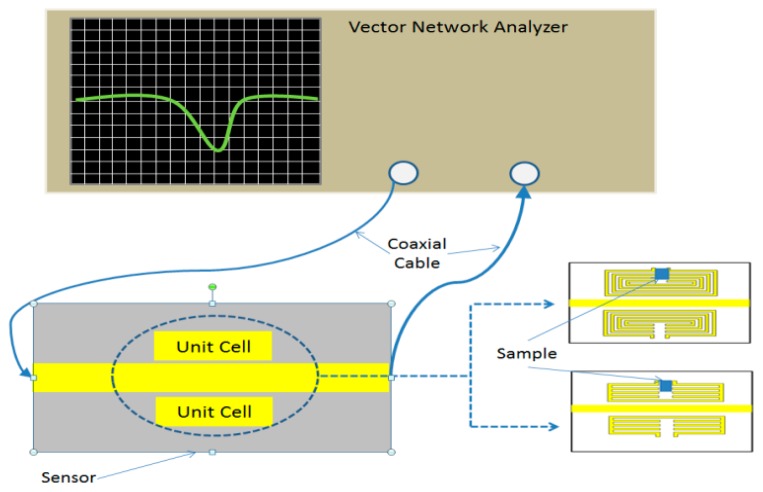
Experimental setup.

The experimental setup is outlined in Figure 7. During the measurement, the response of the sensor is monitored and recorded upon loading with the variation sample under test. Here, the gap capacitance of the proposed sensor is compromised by introducing sample materials in the gap as shown in Figure 7.

## 4. Measurement and Discussions

### 4.1. Sensor Analysis

**Figure 8.**
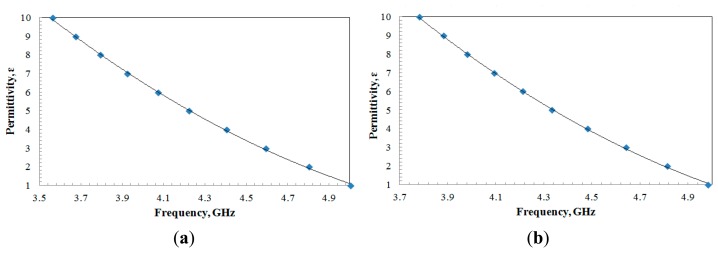
Sample permittivity as a function of resonance frequency for (**a**) aligned-gap multi-ring; (**b**) centered-gap sensor.

To visualize the design for sensing applications, the sensors were loaded and simulated with various dielectric samples from 1 to 10. The variation of results can be observed through Figure 8a,b for both types of sensor. From the results obtain, the resonance frequency is shifted down according to the increasing value of dielectric constant of the samples due to the higher capacitance value when a dielectric material is introduced. As a result, according to Equation (1), the higher the capacitance, the lower the resonance frequency will be. Since the shifts of resonance frequency are used as the data that are related to the permittivity sample, an expression for the relationship between frequency, *f* and permittivity, *ε* of the sample is required. Therefore, an expression for the aligned-gap sensor and centered-gap sensor can be modeled by using a curve fitting method based on the data presented in Figure 8a,b and the polynomials presented in the figure are obtained as follows:

(8) $$\varepsilon =1.62f^{2}–19.99f+60.54$$

(9) $$\varepsilon =2.306f^{2}–27.61f+81.38$$

**Figure 9.**
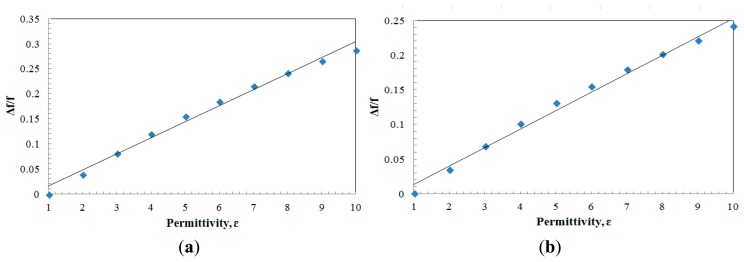
Relative shift (*Δf/f_0_*) *versus* sample permittivity for (**a**) aligned-gap multi-ring; (**b**) centered-gap sensor.

Generally, the sensitivity of the sensor can be represented through the relative shift of measured frequency toward resonance frequency, *Δf/f_0_*. Figure 9a,b plot the relative shift of frequencies as a function of sample's permittivity, *ε_r_*, for each sensor. Thus, from the simulation, the sensor demonstrates a sensitivity of 0.032/*ε_r_* for the aligned-gap sensor while it is 0.026/*ε_r_* for the Centered-gap sensor.

### 4.2. Sensor Experimental Validation

To validate the proposed sensors and estimate their potential in dielectric sensing, the resonance frequency as well as the resonance frequency shift is observed when various dielectric samples were introduced at one of the split gaps of the sensor. In both sensor designs, one side of the resonator is used for detecting the sample while the other side provides the reference frequency. Various samples such as Duroid RT 5880 [27], Rexolite [28], FR4 [29] and plexiglass [27] were put onto the split gap area of sensor. For each design, three sensors were fabricated for reproducibility measurements and each measurement was repeated three times. Initially, baseline measurements were acquired for each new fabricated sample to obtain the relationship between the frequency shift and the dielectric value.

**Figure 10.**
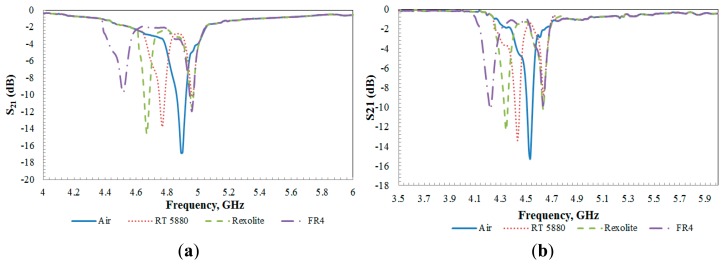
Measured magnitude of S_21_ in dB of solid sample for (**a**) aligned-gap multi-ring; (**b**) centered-gap sensor.

Upon loading, the electric field interacts with the sample, which will cause a shift in the resonant frequency. From the experiment conducted for both sensors, the resonance frequency is shifted to lower frequency with increasing the permittivity value of the sample while the reference frequency is maintained close to constant. Generally, the coupling capacitance between the rings and the gap capacitance both change after adding a sample. These changes are proportional to the permittivity of the dielectric sample. In addition, the variation of the dip depends on the loss tangent of the sample material. Higher sample dielectric loss will contribute to a smaller dip of S_21_ magnitude. Through the observation of results, as predicted in simulations, increasing the sample permittivity reduces the resonance frequency. The difference between theoretical and measured results is presumably caused by manual sample deposition, which results in a slight difference in the sample location. The results given in Figure 10a,b have revealed that the aligned-gap sensor gave a better observable readout shift compared to centered-gap sensor.

**Figure 11.**
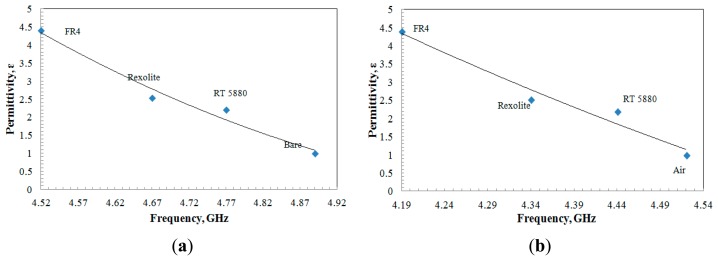
Sample permittivity, *ε_r_ versus* resonant frequency, *f* for (**a**) aligned-gap multi-ring; (**b**) centered-gap sensor.

In order to extract the permittivity value of the sample material, three samples such as air, Duroid RT 5880 and Rexolite were used as calibration samples of which the dielectrics are well known, to obtain the relation between the permittivity, *ε* and the resonance frequency, *f* of the sensor as presented in Figure 11a,b. Three measurements were taken for each calibration sample, and the average values were used in curve fitting method. Thus by using data presented in Figure 11a,b, a polynomial expression for the aligned-gap sensor and centered-gap sensor are obtained as follows:

(10) $$\varepsilon =7.243*f^{2}-76.96*f+204.21$$

(11) $$\varepsilon =3.715*f^{2}-42.04*f+115.27$$

These equations can be employed to determine the dielectric constant of the unknown material under test. Table 4 summarized the experimental results and the extracted value of dielectric properties for each material under test according to the above polynomial equations.

**Figure 12.**
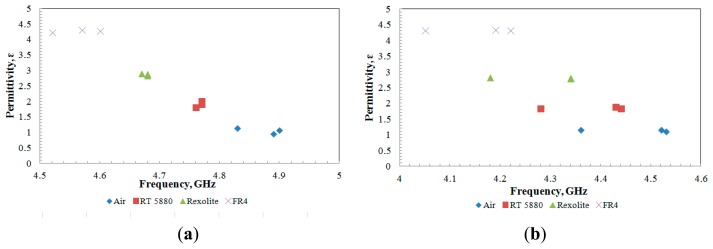
Sensor measurement for three types of sensor for (**a**) aligned-gap multi-ring; (**b**) centered-gap sensor.

Figure 12 shows the reproducibility of the fabricated sensor in measuring the dielectric value of the samples. From the data plotted in Figure 12, the differences found between fabricated sensors were less than 10% for the aligned gap sensor and 5% for the centered gap sensor. It is believed that the small discrepancies are acceptable due to the reason of fabrication tolerance.

**Figure 13.**
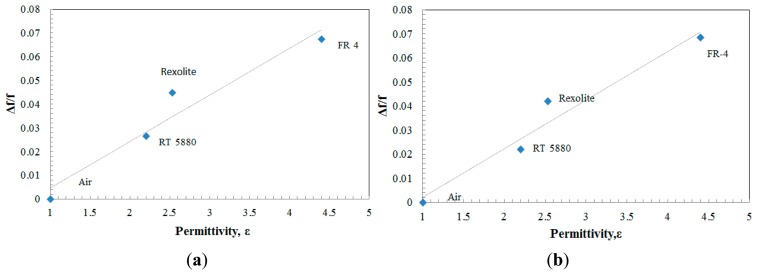
Relative shift (*Δf/f_0_*) *versus* sample permittivity for (**a**) aligned-gap multi-ring; (**b**) centered-gap sensor.

As mentioned in the previous subsection the resonance frequency is considered as an effective microwave parameter of the SRR used as a sensor. Generally, the sensitivity of the sensor related to the resonance frequency can be expressed in a form of *Δf/f_0_*.To demonstrate the high sensitivity of the proposed sensor, an observation of change in the resonant frequency is recorded and tabulated as in Figure 13. From the plotted graph, the sensors demonstrated a sensitivity of 0.019/*ε_r_* for the aligned-gap sensor while it was 0.020/*ε_r_* for the centered-gap sensor.

## 5. Conclusions

This paper presents two designs of microwave sensors based metamaterials, namely the aligned-gap multi split ring resonator and centered-gap multi split ring resonator, for dielectric sensing purposes. It is believed that this structure can also provide greater sensitivity by introducing a sharper and deeper dip which contributes to a high *Q*-factor compared to the conventional designs of SRRs. The simulated and measured results evidently proved that the proposed sensors provide high *Q*-factor and higher concentration of electric field between the extended length of the split gaps which may enhance the sensitivity of the sensor itself.

The aligned-gap and centered-gap sensing probes were coupled to a microstrip line on both sides to realize bandstop properties. The operation of the sensors is based on the shift of the transmission coefficient magnitude S_21_ as a function of the permittivity of a loaded sample. These structures were verified through simulation by introducing in the resonator dielectric samples having different dielectric properties. Once the designs were verified, the proposed structures were fabricated and tested with several types of solid sample as a material under test. From the results obtained, as the permittivity of the sample increased, the frequency shows an obvious minimum shift by 2% from the resonance frequency. It has been demonstrated from the experimental activity conducted, that the aligned-gap and centered-gap sensors are promising candidates to be adopted as transducers in biomolecular sensing applications.

## Figures and Tables

**Table 1.** SRR dimensions.

**Parameters**	**Dimensions (mm)**	

	***Aligned-Gap***	***Centered-Gap***
**Substrate Width, *W***	35.000	
**Substrate Length, *L***	14.000	
**Substrate Thickness, *h***	0.787	
**Unit Cell width, *W****_1_*	9.900	
**Unit Cell height, *L****_1_*	3.300	2.100
**Ring width, *W****_2_*	0.300	
**Ring Gap, *G****_1_*	0.300	
**Split Gap, *G****_2_*	1.000	
**Transmission line width, *W****_tx_*	2.445	
**Gap between SRR and Transmission line, *G****_3_*	0.200	

**Table 2.** Resonance frequency with ranges of the negative permeability for the aligned-gap and centered-gap devices.

**Sensor Type**	**Resonance Frequency(GHz)**	**Frequency Range of Negative Permeability (GHz)**
**Aligned-Gap**	5.00	4.89–5.10
**Centered-Gap**	4.98	4.89–5.12

**Table 3.** Simulated and measured resonant frequency, Q factor and S_21_ magnitude in dB.

**Type of Sensor**	**Resonance Frequency (GHz)**		**Q Factor**	**Magnitude of S_21_ (dB)**	
	
	***Simulation***	***Measurement***		***Simulation***	***Measurement***
Aligned-Gap	5	4.90	240	−25.60	−16.79
Centered-Gap	4.98	4.53	150	−23.30	−15.26

**Table 4.** Summarized experimental results for the aligned-gap and centered-gap sensor.

**Aligned Gap Sensor**	**Air (Calibrate Sample)**	**Duroid (Calibrate Sample)**	**Rexolite (Calibrate Sample)**	**FR-4 (Calibrate Sample)**	**Pexiglass (Unknown Sample)**
Reference Permittivity, *ε_r_*	1	2.2	2.53	4.4	2.6

Aligned-Gap MSRR Sensor					

Calculated Permittivity, *ε_r_*	1.08	1.92	2.78	4.33	2.97
Frequency, *f* (GHz)	4.90	4.77	4.67	4.52	4.65

Centered-Gap MSRR Sensor					

Calculated Permittivity, *ε_r_*	1.14	1.85	2.79	4.34	2.99
Frequency, *f* (GHz)	4.53	4.44	4.34	4.19	4.32
